# Cases Illustrating Different Degrees of Susceptibility to Electricity

**Published:** 1875-06

**Authors:** George M. Bread


### CASES ILLUSTRATING DIFFERENT DEGREES OF SUSCEPTIBILITY TO ELECTRICITY.

BY GEORGE M. BREAD, M. D.

I apply the term *electro-susceptibility* to that peculiarity of the system that makes it capable of being affected for good or ill by electricity. Electro-susceptibility refers to the distant and the permanent, as well as to the direct and immediate effects of electrical treatment, and therefore differs from electro-sensibility, which is restricted mainly to the sensitiveness of the body, to electricity as evidenced during the application.

Electro-susceptibility may be subdivided into farado-susceptibility and galvano-susceptibility ; some are very susceptible to the influence of the faradic and not to the galvanic, and *vice versa;* and some are very susceptible to both currents.

We recognize these different orders and modifications of susceptibility to various medicines in daily use. Thus there are many—and their number seems to be increasing—who are very susceptible to opium in any form or preparation ; they cannot take even a small amount without suffering headache and nausea, and they are kept awake rather than made sleepy by it. Again, there are some who can bear and be benefited by certain preparations of opium, but not by others. Similarly, hydrate of chloral does better for some; bromide of potassium for others ; and there are those who can be made to sleep only by a combination of the two remedies.

It is impossible to tell from the appearance of any patient whether he will or will not be susceptible to opium, or bromide of potassium, or alcohol; still less can we tell from personal appearance which form of opium will best and which will worst suit him, or whether bromide of potasium or hydrate of chloral will give him the best relief from insomnia.

This general law, however, holds that the nervous and delicate are more susceptible to all these powerful remedies than the phlegmatic and the strong, and that among the higher and brain-working classes smaller doses must be used and greater caution is requisite than among the lower and muscle-working classes. But both ways the exceptions are many. There are strong, large, and burly men who cannot bear opium or alcohol, but very little of opium, and so on; and contrariwise, there are sensitive and exquisitely delicate women on whom alcohol or opium makes little impression. The range and variety of idiosyncrasy in these respects are infinite and mysterious.

In their electro-susceptibility patients similarly vary. As a general law, the higher classes bear less of electricity than the lower class: women less than men; Americans less than Europeans; Fifth Avenue must be treated more tenderly than Five Points.—And yet the exceptions of this general law are numerous and striking; there are sensitive and feeble women in the last stages, it may be, of some severe disease, who can bear enormous doses of electricity, and there are sturdy men who can drink whisky by the tumblerful and smoke all day, and who can easily be made miserable by a mild current applied for less than five minutes.

For some mysterious cause that may never be explained, some patients who are made worse by the faradic current in the method of general faradization, are benefited by the galvanic current in the method of central galvanization.

In some cases excellent results are obtained by alternating these methods, or by combining them in the method of galvano-faradization. In some cases I have seen advantage from using both general faradization and central galvanization at the same sitting, not simultaneously, but in succession.

In regard to this matter of susceptibility to electricity, there are three facts of great interest as well as of practical importance.

*First*, Children are less susceptible to electricity than adults.—In experiments made during the past two years I have shown that infants of both sexes, at an extiemely early age, from a few weeks or months, oft-times can bear almost if not quite as much electrical treatment, faradic or galvanic, central, general or local applications, as the average adult. The immediate effect of electrical treatment of infants is to produce sleep and allay irritation, just as in adults, but more positively; the permanent effects are increase of liveliness and vigor, improvement in desire and capacity for nursing and taking food, and these permanent effects are not so often, as in adults, complicated with unpleasant effects indicating special susceptibility, which I judge that in the infantine constitution there is some quality, or lack of quality, that renders it less susceptible to the electrical force than the constitution of adults. The one practical difficulty of making full comparison in this respect, on account of the slight intelligence of children, and their inability to observe or express their feelings, I admit of course; but when the electrical treatment produces calmness and repose in children, as in adults, it is usually, if not always, safe just to assume that all its effects are good, and only good, and that, even if the special disease is not cured, the patient will not be harmed, and, in a general way, at least, will be benefited.

*Secondly,* Those who are at first unpleasantly susceptible to electricity may in time become so tolerant of it as to be benefited by it.

It is important, therefore, not to be discouraged when at the beginning of a course of treament various unpleasant symptoms occur; but to persevere, making the applications short, mild, and giving long intervals.

*Thirdly,* A limited number of patients in the higher walks of life can never acquire a tolerance of electricity, however cautiously it may be given, or however long the intervals between the applications. To persevere in treating such persons, after full and fair trial by very mild currents carefully given, by long intervals, is a blunder. The disagreeable effects that follow the applications, even on this class of patients, are, however, usually of a temporary character.

*Fourthly,* There are occasionally found those who are but slightly susceptible to electricity, who can bear long and strong applications, but who, notwithstanding, are not benefited by it. Of the various class of patients with whom we have to deal, this is the most provoking; they can be treated infinitely, and without showing any good effects of treatment; they become neither better nor worse, and no amount of perseverance seems to have any reward.

The following case shows that a delicate person may be unpleasantly susceptible to the faradic current, may bear good doses and be benefited by the galvanic current,—in other words, there may be great farado-susceptibility, and only average galvano-susceptibility.

Case I.—Miss P—, an unmarried lady of about 38 years of age, had been paralyzed for some years in the lower limbs, and withal had passed through a terrific storm of hysterical symptoms, general hyperæsthesia, insomnia, trembling, trepidation, debility in the extreme, morbid fear, and so on.

She had been treated at one time by general faradization, without important benefit. When I first saw her the symptoms were partial paralysis of the lower limbs, and great pain in the dorsal and lumbar region of the back on attempting to walk, and some insomnia. I treated her at first by local faradization of the paralyzed muscles, but this, though carefully used, seemed to irritate. I tested this question several times, and assured myself that it was not a coincidence—that she was susceptible more than is wont to the faradic current. I then tried the galvanic current centrally and locally, and found that she could bear this well and frequently, and improved under it rapidly in all her symptoms. I found that she could bear easily central galvanization with quite strong currents, applied for a considerable time, and that such treatment was followed always, or almost always by a relief of nervousness and a disposition to sleep, and by mitigation of the hysterical pains in the back and limbs. She improved so far that she could walk about, and for a considerable distance, without the need of the mechanical support that she had formerly used.

The following case illustrates the important fact that one may be very susceptible to electricity, may indeed be harmed by a severe shock, and yet be benefited by careful management, and appreciate on the average all the well-known tonic effects of electrical treatment.

Case II.—A young physician had been troubled for some time with neurasthenia and hypochondriasis. It was found by trial that he was quite susceptible to electricity, and it was necessary to be very moderate in the doses. Central galvanization was the method chiefly used, and under this treatment, given at considerable intervals and in conjunction with internal medication, there was an improvement in his symptoms that promised to be and has been permanent for one yeear at least; but on one occasion I chanced to give him a moderately severe shock through the head, by interrupting the current when the positive pole was over the cranial centre and the negative pole at the pit of the stomach, and as a result he was put back in his progress towards recovery by at least a month. Possibly the patient, who is a medical gentleman of intelligence, may have overestimated the evil effects of this single shock, and the mind may have acted on the body, and may have reinforced a slight genuine evil by a great imagined evil; but I will admit that he was right in his opinion, and that his temporary relapse was caused by the interruption of the current through the head, and by no other agency. Such results are, however, excessively rare. I have never met with another just like it. The evil symptoms that follow such shock through the head and neck usually pass away in a few moments or hours, or at least in a day or two at most, and the patient returns to his normal condition.—Very few indeed are at all injured by them. In the present case the treatment was continued after an interval, and the gentleman is, I believe, permanently better.

Case III.—A young man, a mechanic, had been troubled with impotence for several years, the result of various excesses. His impotence was not absolute, being rather a diminution than abolition of virile power, and uncertainty in the execution.

I found that this patient could bear, without any perceptible ill effect, very powerful currents, galvanic or faradic, applied for a long time, externally or internally. In using the urethral electrode I found that a certain portion of the canal about one inch in length had so little of electro-sensibility that strong faradization gave slight pain.

In spite of this unwonted tolerance of electricity the patient improved but very little. In the majority of cases impotence and spermatorrhœa are very much benefited by judicious electrical treatments, and in some instances the results are most gratifying; but in this case perseverance was rewarded by only a little increase of power.

The case is of interest mainly as illustrating that it is possible to be very tolerant of electricity in any form, or however administered, without showing any effect of electrical treatment. The tolerance gives no advantage. The prognosis is better when there is great susceptibility—when there is, so to speak, something to work on—something to be done in the way of building up a sensitive and nervous constitution.

CASE IV.—A widow lady, about thirty years of age, had all her life been nervous ; among very many other difficulties, had suffered from obstinate and long-standing prurigo, and podalgia, or aching of the feet, with paresis of the extensors of the legs. I treated her by central galvanization, and also by local faradization and galvanization of the paretic muscles of the aching feet. The result has been remarkable for the temporary relief of the prurigo, and the strengthening of the extensor muscles. Her catamenia came in the middle of the treatment, and lasted in all seventeen days; they were also quite profuse, and weakened her not a little. It was clear that the electricity was the cause, for previously she had been annoyed by amenorrhœa. The case was interesting also as showing that electrization of the lower limbs and feet may, by reflex action, have a most powerful effect on the pelvic organs. At no time were the applications made through or over the uterus or ovaries. At one time when the courses had apparently stopped, electrical treatment of the feet was resumed, and at once the discharge reappeared, and for several days the flow was profuse.

In spite of these untoward experiences the lady persevered, and was rewarded by improvement of all her symptoms, of a most remarkable character.

CASE V.—The wife of a physician was treated by me for rheumatism affecting especially the shoulders, and the applications were made mainly to the shoulders and arms, and mild currents were used. The effect was to bring on a very profuse and prolonged menstruation, by which the treatment was interrupted, and it was not resumed.

In this case, as in the preceding one, the patient might have acquired a certain tolerance of electricity and been benefited had she persevered.

Some of the reflex effects of electricity are very interesting and suggestive.

Case VI.—A gentleman, who has for years been troubled with hyperæsthesia of the left hand and fingers on the palmar surface, appears to be very susceptible to either current, to whatever part of the body it may be applied. A very mild galvanic current causes a sense of numbness and rigidity in the affected arm and hand, and sometimes also induces a tingling or pricking in theother hand and arm.

These abnormal feelings, with many variations, may continue for several hours or days following the application. In spite of this impressibility, the appetite of the patient appears to improve under the treatment, although the hyperæsthesia, which was of a troublesome character, remains the same.

Case VII.—A lady that was treated by galvanization through the lumbar region and bowels, remarked that when the electrode was placed over the middle of the lumbar vertebræ, a peculiar sention was felt at the crown of the head—the cranial centre, as I have termed it—where pain and burning sensations are so often experienced by women suffering from the uterine disorders, and the place where the positive pole is rested in the method of central galvanization. The reflex action takes place, we may suppose, through the occipital nerves.

This same patient, when treated at the anus, sometimes experienced a slight thrill or transient numbness over the arms and hands. The effects were noticed when feeble galvanic currents were used, and the electrodes were placed less than a quarter of an inch from each other. The effects must then be purely reflex.

I suspect that in surgical as well as in medical disease this element of the susceptibility of the patient to the electricity is to be considered and is of importance in the prognosis. It is observed that the pain accompanying various forms of malignant tumors is frequently very much relieved by local galvanization; this relief may appear in the midst of an application, and may continue for hours, perhaps for a day or two.

With this relief of pain there may be an apparent arrest of the growth; even though it does not grow any smaller it appears to stop growing larger, or at least increases more slowly than before treatment. If the tumor be ulcerating, the ulcerated surface, if treated, may put on a healtheir appearance, and the quantity and quality of the discharge may be very greatly modified. But there are malignant tumors, which to external appearance at least, are not of the ugliest type, and which are painful, but the pain is but little relieved by galvanic treatment with or without the needles, and there is no arrest of the growth. Whether the different behavior of these tumors depends on their intimate structure, pathologists must determine; but the query may justly arise whether the temperament of the patient--the electro-susceptibility—may not have something to do with these opposite results of treatment by electricity. It may be that if a tumor over which electricity had no power were transferred to another person of a diferent temperamen, the result would be more favorable.

I have recently treated two cases of tumor of the breast, with a view to relief of pain. In one case, the more advanced and most malignant in appearance, the relief of pain, and of the uneasiness that is sometimes worse than pain, has been most grateful, and the tumor, though it had long been ulcerating, has made little advance. Withal, the general vigor of the patient has improved under central applications. She experiences no evil effects from even strong and prolonged treatment; her temperament without regard to any disease from which she may be suffering is adapted for electrization. At one time she suffered from rheumatism, which was promptly and permanently relieved by galvanization.

In the other—a lady younger than the other, and fully as strong —a tumor of the breast, similar in the general character and malignancy, but smaller and taken much earlier, has not been benefited by electrical treatment; the growth was not only not arrested, but went on rapidly. When central applications were made the patient gave evidences of unusual susceptibility to electricity. Is it not possible that if these two patients could exchange their tumors, the results of treatment might be reversed.

Some cases are susceptible in a most interesting way to the hypnotic effects of electricity. I once treated by central galvanization a young lady who almost always closed her eyes, began to nod in less than two minutes after the positive electrode was placed over the region of the sympathetic and pneumogastric, at the anterior border of the sterno-cleido-mastoid muscle. If the current were allowed to run for several minutes the patient would drop fast to sleep, but of course was easily awakened. Rapid hypnotic effects were produced also whenever the current was applied in the back or around the neck in any direction.

I have recently treated another case, where similar, though less decided hypnotic effects were obtained.

In some cases the susceptibility of patients to electricity shows itself very slowly—perhaps not until a day or two following an application. Such cases are very illusory; it takes close watching on the part of the physician to detect this peculiarity. Just after a séance, and very likely for several hours following each séance, the patient may feel as well as ever—possibly a little relieved—but on the evening of that day, or on the following day, the unpleasant symptoms may appear.

Cases of this kind need not be abandoned; they may be positively and permanently strengthened by cautious and faithful treatment; but they must be treated warily—sometimes by long intervals, possibly several days between the applications.—*The Medical Record.*

